# First person – Christian Gonzalez

**DOI:** 10.1242/dmm.052691

**Published:** 2025-11-26

## Abstract

First Person is a series of interviews with the first authors of a selection of papers published in Disease Models & Mechanisms, helping researchers promote themselves alongside their papers. Christian Gonzalez is first author on ‘ Degenerating intervertebral discs in the streptozotocin-high-fat diet model of type 2 diabetes show extensive inflammation’, published in DMM. Christian is a PhD student in the lab of Simon Tang at Washington University in St. Louis, St. Louis, MO, USA, investigating how chronic inflammation and diabetes promote intervertebral disc degeneration and lower-back pain.


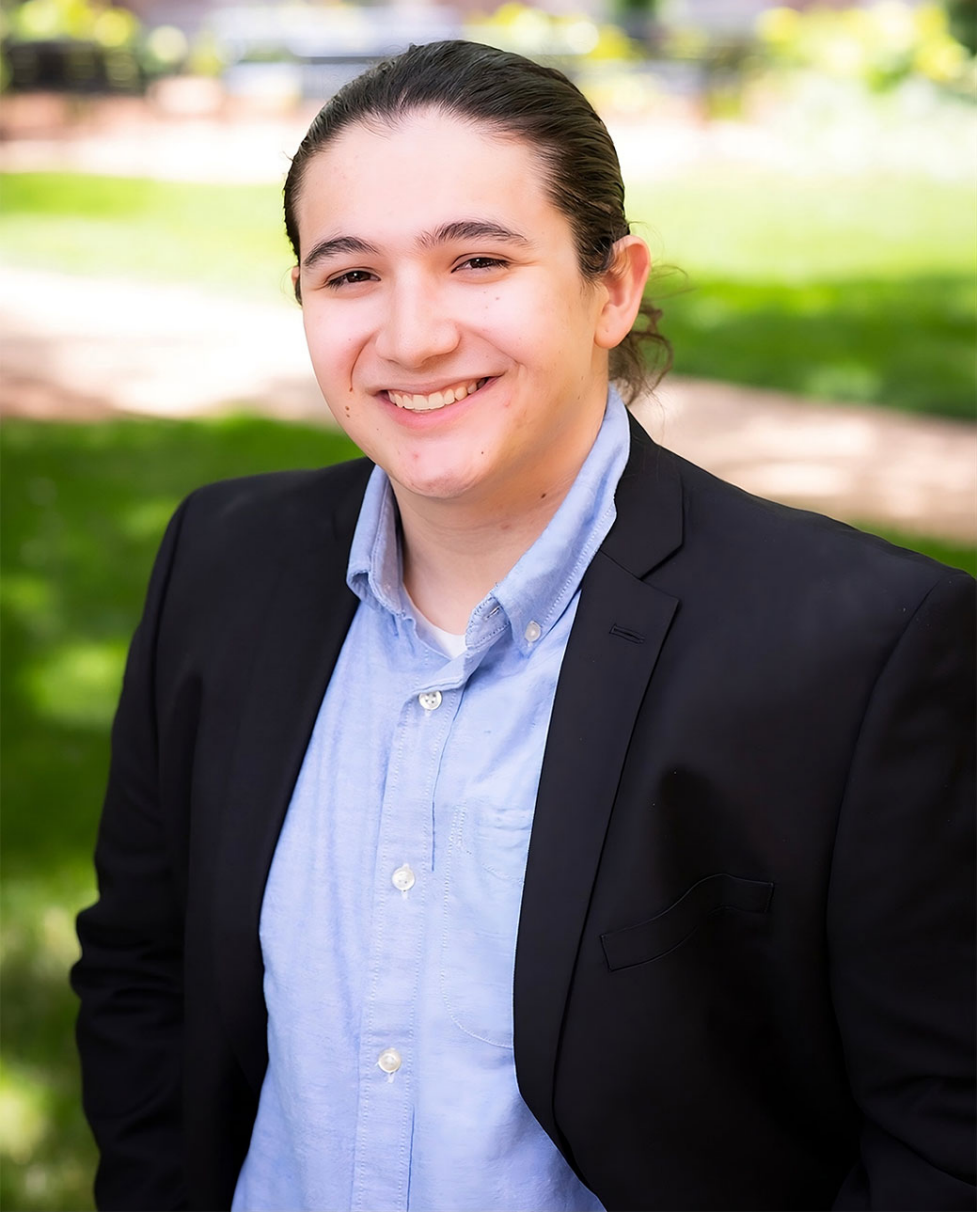


**Christian Gonzalez**

**Who or what inspired you to become a scientist?**

My earliest and most enduring inspiration to pursue science was my mother. She suffered a disabling spinal injury early in life that required multiple surgeries and left her with chronic pain. Watching her navigate the challenges of daily life with resilience but also with ongoing suffering gave me a sense of urgency to devote myself to research. I grew up asking questions about why such conditions happen, why they persist despite medical care and what could be done differently to improve outcomes. Over time, these questions transformed into a genuine passion for biomedical science. I came to see science not just as an academic pursuit but as a means of contributing to solutions for people like my mother. Her experience inspired me to focus on musculoskeletal and pain-related conditions, where advances could significantly change quality of life. Even today, whenever experiments get tough or setbacks occur, I think of her journey, which keeps me motivated. In this way, my career path has been shaped as much by personal experience as by scientific curiosity.

… our work underscores the need to treat diabetes as more than a metabolic condition, recognizing its wide-reaching effects on the spine

**What is the main question or challenge in disease biology you are addressing in this paper? How did you go about investigating your question or challenge?**

The central question we are addressing is how different models of diabetes impact the progression of intervertebral disc degeneration and associated inflammation. Diabetes is a highly complex disease that manifests differently depending on genetic and metabolic factors, so a single model cannot fully capture the range of pathology. To approach this, we compared two commonly used mouse models: the db/db genetic model and the STZ-HFD chemically induced model. These models each mimic distinct aspects of human type 2 diabetes, allowing us to examine whether their influence on spinal degeneration is similar or divergent. We designed experiments that focused on both structural and molecular outcomes, carefully quantifying disc changes and measuring inflammatory markers. This dual strategy gave us a way to integrate anatomical and cellular perspectives on disease progression. By taking this comparative approach, we were able to uncover differences that might otherwise remain hidden if we relied on only one model. Ultimately, this investigation helps clarify how diabetes drives degeneration and informs which models may be most appropriate for future translational studies.

**How would you explain the main findings of your paper to non-scientific family and friends?**

Back pain is something almost everyone experiences at some point, and for many people it becomes a long-term problem. One reason this happens is that the discs in the spine (the cushion-like structures between the bones) start to break down as we age or when disease makes them more fragile. In this study, we looked at how diabetes makes that breakdown worse. We used mice as a way to study the condition more closely, because we can control the environment and track changes in ways we can't with people. What we found is that diabetes can increase inflammation and that this process can speed up damage in the discs. So, in simpler terms: having diabetes can make back problems worse because it changes how the body handles damage and healing. These findings might help explain why people with diabetes are more likely to experience chronic back pain. They also suggest new ideas for treatments that focus on reducing this added layer of inflammation.

**What are the potential implications of these results for disease biology and the possible impact on patients?**

The implications of this study are meaningful both for basic science and for future clinical care. On the biological side, our findings highlight that not all forms of diabetes affect spinal health in the same way, which means disease models must be chosen carefully in research. By identifying patterns of inflammation and degeneration specific to each model, we gain a deeper understanding of the cellular mechanisms that make diabetic patients more vulnerable to disc-related problems. For patients, this could eventually lead to new therapies that target the inflammatory pathways we identified. For example, drugs that reduce specific inflammatory signals may help slow or even prevent disc degeneration in people with diabetes. In addition, our results may encourage clinicians to screen diabetic patients more carefully for spinal health, catching problems earlier. Beyond the spine, this study contributes to a larger body of evidence showing how systemic diseases like diabetes can influence musculoskeletal health. Overall, our work underscores the need to treat diabetes as more than a metabolic condition, recognizing its wide-reaching effects on the spine.

**Figure:**
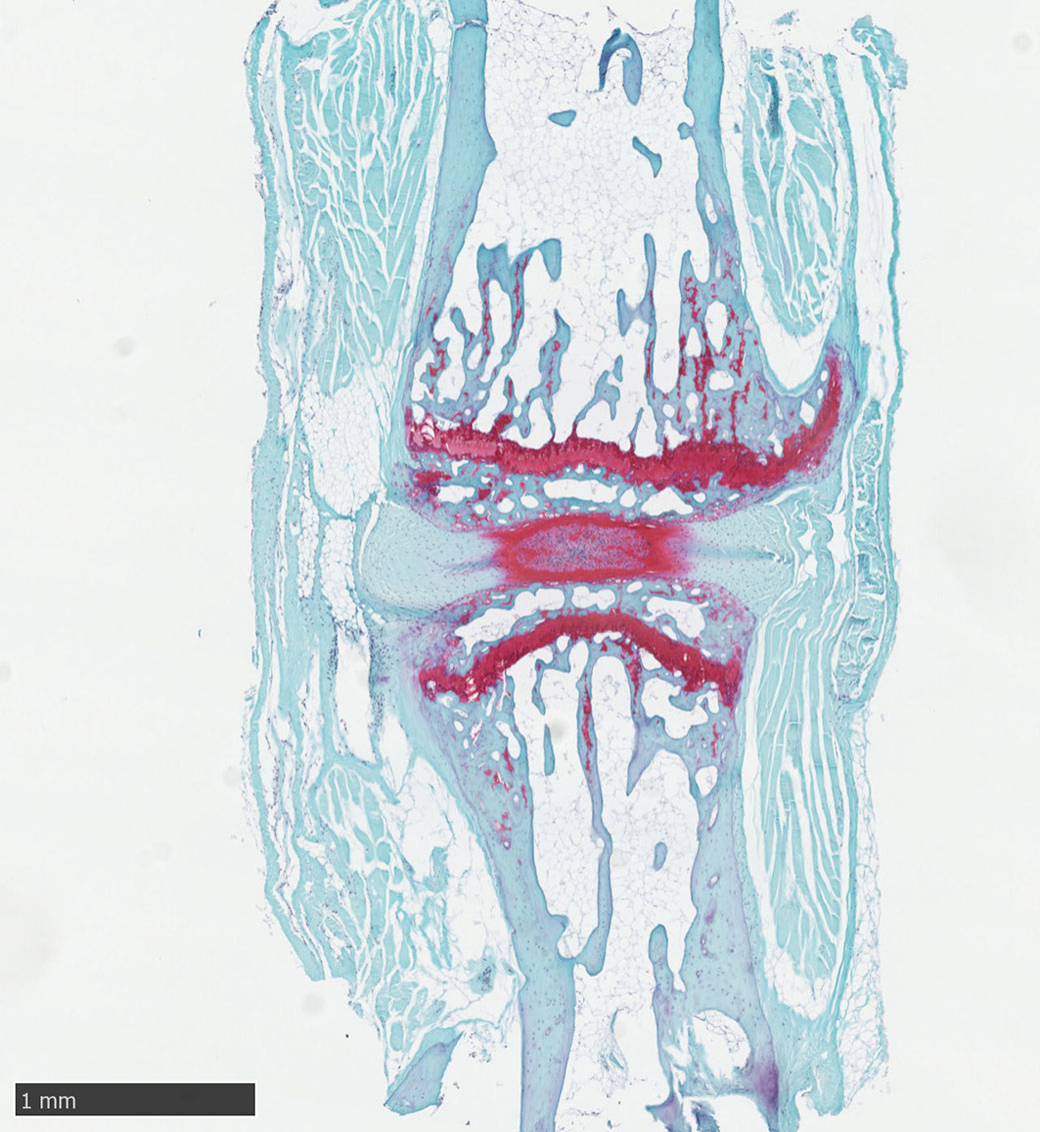
Our work provides early evidence that, in the streptozotocin-high-fat-diet mouse model of type 2 diabetes, the intervertebral disc shows early signs of degeneration in histopathological analysis.

**Why did you choose DMM for your paper?**

We chose DMM because it has a strong history of publishing high-quality work that bridges fundamental biology with disease relevance. The journal is particularly known for advancing studies that use animal models to shed light on mechanisms of human disease, which is exactly the goal of our research. Our project required a platform that appreciates the importance of comparing models and highlighting their translational value, and DMM provides that audience. Furthermore, DMM has published a number of impactful papers on both diabetes and musculoskeletal disease, making it an ideal venue for our work. The readership is highly interdisciplinary, which means our findings can reach scientists focused on metabolism, inflammation and orthopaedics alike. This breadth is essential because the intersections of diabetes and back pain cross multiple fields. We also wanted a journal with strong peer review standards to ensure our work contributes meaningfully to the literature. For these reasons, we believe DMM is the best fit for communicating our findings and their broader implications.

… without clear systems, data can quickly become overwhelming

**Given your current role, what challenges do you face and what changes could improve the professional lives of other scientists in this role?**

As a late-stage graduate student, one of the biggest challenges I face is balancing the increasing complexity of experiments with the growing responsibility of managing projects independently. At this stage, the science itself becomes more ambitious, often involving multiple models, techniques and collaborators. The pressure to publish and complete a dissertation adds another layer of stress. Staying organized is absolutely critical, because without clear systems, data can quickly become overwhelming. Another challenge is managing time effectively, since writing, experiments, mentoring junior students and professional development all compete for attention. In addition, funding pressures and the uncertainty of career transitions can create significant anxiety. To improve the lives of scientists in this role, institutions could place a stronger emphasis on mentorship, mental health resources and training in project management. Providing graduate students with tools to build organizational skills early would make later stages of training smoother. Overall, the path becomes more manageable when support systems are in place, and fostering these could help ensure young scientists thrive rather than burn out.

**What's next for you?**

I plan on seeking an industry role that allows me to fully utilize the many computational, experimental and organizational skills I've accumulated over my years in training. Ideally, I would like to advance therapeutics addressing inflammatory disorders, pain and degeneration.

**Tell us something interesting about yourself that wouldn't be on your CV**

I love cooking! I first started with classical French and Italian cooking, but I've spent years exploring different foods from around the world. I run an Instagram page where I journal about the foods I've made over the years.
